# Pancreatitis-Associated Protein in Neonatal Screening for Cystic Fibrosis: Strengths and Weaknesses

**DOI:** 10.3390/ijns6020028

**Published:** 2020-03-30

**Authors:** Olaf Sommerburg, Jutta Hammermann

**Affiliations:** 1 Division of Pediatric Pulmonology & Allergy and Cystic Fibrosis Center, Department of Pediatrics III, University of Heidelberg, Im Neuenheimer Feld 430, D-69120 Heidelberg, Germany; 2 Translational Lung Research Center Heidelberg (TLRC), Member of the German Center for Lung Research (DZL), Im Neuenheimer Feld 350, D-69120 Heidelberg, Germany; 3 Pediatric Department, University Hospital of Dresden, Fetscherstr. 74, D-01307 Dresden, Germany; Jutta.Hammermann@uniklinikum-dresden.de

**Keywords:** cystic fibrosis, newborn screening, biochemical screening, pancreatitis associated protein, immunoreactive trypsinogen

## Abstract

There are currently four countries and one local region in Europe that use PAP in their newborn screening programme. The first country to employ PAP at a national level was the Netherlands, which started using IRT/PAP/DNA/EGA in 2011. Germany followed in 2016 with a slightly different IRT/PAP/DNA strategy. Portugal also started in 2016, but with an IRT/PAP/IRT programme, and in 2017, Austria changed its IRT/IRT protocol to an IRT/PAP/IRT program. In 2018, Catalonia started to use an IRT/PAP/IRT/DNA strategy. The strengths of PAP are the avoidance of carrier detection and a lower detection rate of CFSPID. PAP seems to have advantages in detecting CF in ethnically-diverse populations, as it is a biochemical approach to screening, which looks for pancreatic injury. Compared to an IRT/IRT protocol, an IRT/PAP protocol leads to earlier diagnoses. While PAP can be assessed with the same screening card as the first IRT, the second IRT in an IRT/IRT protocol requires a second heel prick around the 21st day of the patient’s life. However, IRT/PAP has two main weaknesses. First, an IRT/PAP protocol seems to have a lower sensitivity compared to a well-functioning IRT/DNA protocol, and second, IRT/PAP that is performed as a purely biochemical protocol has a very low positive predictive value. However, if the advantages of PAP are to be exploited, a combination of IRT/PAP with genetic screening or a second IRT as a third tier could be an alternative for a sufficiently performing CF-NBS protocol.

## 1. Introduction

Cystic Fibrosis Newborn screening (CF NBS) is widely accepted, but there is no universal screening strategy [1]. All programs start with a measurement of immunoreactive trypsinogen (IRT) in dried blood spots. As the second tier, a repeat measurement of the IRT concentration can be performed at the age of 2–3 weeks, but in the most common CF NBS protocols, IRT measurement as the first tier are combined with the search for population-specific *CFTR* mutations, which provides good sensitivity and specificity [2]. However, the use of *CFTR* mutation analysis is also associated with a few unsolved problems. For example, the detection of healthy carriers and of infants in whom the diagnosis of CF is inconclusive (CFSPID) is not the goal of CF NBS. Furthermore, with increasing migration in the world and the mixing of different ethnic groups, especially in big cities, there is a tendency in countries with genetic CF NBS to increase the number of *CFTR* mutations tested to ensure sufficient sensitivity. This leads to a further increase in the number of carriers and CFSPID. However, this makes information and counselling for families with children with CF, carriers, or CFSPID in these countries increasingly challenging [3,4]. In addition, in countries where informed consent for CF NBS is required, genetic CF NBS can significantly complicate the parental education and consent process.

In 1994, a French group suggested pancreatitis associated protein (PAP) as candidate for a marker for screening CF [5]. PAP is a secretory protein which is not measurable in blood under normal conditions, but which can be detected in high quantities in the context of pancreatic injury [6]. Two pilot studies showed that almost all IRT-negative newborns and most IRT-positive newborns without cystic fibrosis had normal PAP, while PAP was increased in newborns with CF [5,7]. Yet, the increase in PAP observed in newborns is not strictly CF-specific. If the measurement of PAP were used for CF NBS alone, it would have a similarly low specificity as the use of IRT alone. In the first French pilot studies, however, it was found that newborns with CF always had both an increased IRT and an increased PAP; in a further study, it was concluded that both parameters should be evaluated. The aim of this feasibility study was to compare the sensitivity and specificity of the combined measurement of IRT and PAP in the same neonatal population with the screening strategy (IRT/DNA/IRT) used in France at that time [8]. In this study, 204,748 newborns were included; the results published in 2005 showed that the performance of the IRT/PAP strategy was not inferior to that of the IRT/DNA/IRT strategy applied in parallel [8].

## 2. The Evolution of the PAP Kit

It is important to mention right at the beginning that the PAP kit has undergone several changes and improvements since it first appeared. For the data of the first publications on the use of PAP in CF NBS obtained from 1994 to 2003, an ELISA kit using a polyclonal antibody for antigen capture and detection was used [5,8,9]. When Sarles et al. published their lauded paper on the IRT/PAP protocol including recommended cut-offs in 2005 [8], the manufacturer (Dynabio, Marseille, France) had already changed the ELISA kit used for this evaluation, and the original kit, to which the recommendations referred, was no longer available. At that time, a new kit, called “MucoPAP”, was available, which uses monoclonal antibodies to capture and detect antigens. Unfortunately, there were no new recommendations for the cut-off values for this MucoPAP kit to serve as guidelines. Thus, pilot studies that were later conducted in other European countries and which are described below used the cut-off values that were actually set with the previously-marketed kit. The new cut-off recommendations for the MucoPAP kit with the monoclonal antibody were published by Sarles et al., but not before 2014 [10]. In the meantime, however, results from other European pilot studies had been published [11,12,13]. Some had used different cut-off values in their protocols or had used different safety net strategies to ensure sufficient sensitivity [11,12,14]. During the pilot study in the Netherlands, which will be discussed below [12], the researchers realized that the dilution factor recommended in the product description of the manufacturer of the MucoPAP kit for calculating the measured values after comparison with the reference standard was incorrect. After contact with Dynabio, this was officially corrected, but this meant that the originally recommended cut-off values had to be corrected by a factor of 1.67. To avoid further confusion for the reader, we will mention from now on in this review only the values with the corrected dilution factors, but we will add the noncorrected values in parenthesis, if these values were used in the respective original articles (e.g., in [8,11,13]). 

From 2013 onwards, a further version of the PAP-ELISA, the MucoPAP-F-Kit, was available from DynaBio, which uses an alternative readout system. With this kit, the antigen–antibody complexes are detected by a streptavidin–europium conjugate, which serves as fluorescence enhancement solution. This makes it possible to detect highly fluorescent chelates that emit at 620 nm when excited at 337 nm. Compared to measurements with the MucoPAP kit with photometric detection, the MucoPAP-F kit seems to be much more stable and has a higher reproducibility. It is important to note that the cut-off values of PAP measurements with MucoPAP and MucoPAP-F are not directly comparable. 

In 2016, Dynabio launched a new version of its PAP kit with photometric detection, the “MucoPAP II”. The company claimed that this test had a much better intraspot reproducibility of ranges and controls compared to the previous MucoPAP kit, but the calculations from the new ranges were ~1.5 times lower than with the old kit. As a result, the PAP cut-off values had to be changed again, as done for the Austrian CF NBS in 2017.

Unfortunately, the different PAP cut-off values published over the years have meant that publications on the performance of PAP-based CF-NBS protocols are very difficult to compare.

## 3. Description of Selected European Pilot Studies

**Figure 1:**
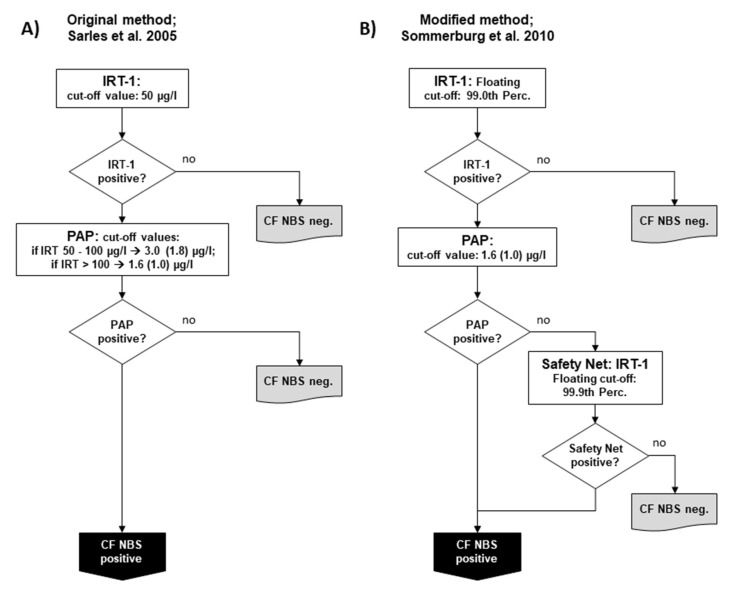
Schemes of the two main variants of the pure biochemical IRT/PAP protocol: (**A**) IRT/PAP protocol published by Sarles et al. 2005 [8] and (**B**) the IRT/PAP-SN protocol with IRT-dependent safety net modified by Sommerburg et al. [11]. Values in parenthesis show the PAP cut-off values as given before correction of the dilution factor by the manufacturer (see explanation in the main Text).

In 2005, Sarles et al. published their study, which demonstrated the feasibility of using PAP in conjunction with IRT [8]. While IRT and PAP were measured in parallel during the study, after an evaluation, the authors proposed a protocol in which IRT is used as the first tier and PAP as the second, which is only performed in case of increased IRT. In this respect, the so-called IRT/PAP protocol was very similar to the IRT/IRT and IRT/DNA protocols known before. In the protocol proposed by Sarles et al., a fixed IRT cut-off value of 50 µg/L was used to ensure sufficient sensitivity. For PAP, two IRT-dependent cut-off values were proposed to reduce the number of newborns with CFSPID and improve the positive predictive value (PPV): If IRT was measured between 50.0–99.9 µg/L, a PAP cut-off value of 3.0 (before correction of the dilution factor 1.8) µg/L should have been applied; if IRT was > 100 µg/L, a PAP cut-off of 1.67 (before correction of the dilution factor 1.0) µg/L should have been used [8] (Figure 1A). This protocol was the starting point for all changes that were later made in other CF NBS protocols based on PAP.

After the publication of this study in 2005, many specialists involved in CF NBS were interested in PAP as a new biochemical parameter and as an alternative to genetic CF screening. Although IRT/DNA protocols became the gold standard for CF NBS in terms of sensitivity and PPV, they had the disadvantages described above. However, if IRT/PAP is used as a pure biochemical protocol, the detection of healthy carriers can be completely avoided. This was the reason why studies were started in several countries around the world in the following years to verify the results of the French study and to adapt the method to local requirements. Unfortunately, not all the results of these studies were published. To the best of our knowledge, data are currently available only from France [10], Germany [11,15], The Netherlands [12,16], Czech Republic [13] and Portugal [17]. 

In 2008, new pilot studies started in the Netherlands and Germany. In the study in the Netherlands, samples from 145,499 newborns were measured using the slightly modified IRT/PAP protocol proposed by Sarles et al. [8], and the results were compared with those of an IRT/DNA/EGA protocol [12]. In the modified IRT/PAP protocol, the IRT cut-off used was set at 60 instead of 50 µg/L. Furthermore, the photometric measurement of the commercially available MucoPAP kit (Dynabio, Marseille, France) was replaced by a flouroimmunoassay using a Streptavidin-Europium tracer for the detection of PAP in a manner that is similar to that later introduced in the MucoPAP-F kit. The two IRT dependent PAP cut-offs were performed as follows: a positive result for PAP was defined if IRT was ≥ 100 µg/L and PAP was ≥ 1.6 µg/L or IRT was ≥ 60 µg/L and PAP was ≥ 3.0 µg/L. In the IRT/DNA protocol, the *CFTR* gene was sequenced (extended gene analysis, EGA) if, in an initial search with a panel of 35 *CFTR* mutations, none or only one *CFTR* mutation was found. In a post hoc analysis, a combination of both strategies (IRT/PAP/DNA(35)/EGA) was shown to be the best compromise for the requirements of the CF NBS program in the Netherlands.

In Germany, separate pilot studies were started in 2008 in two NBS centres (Dresden and Heidelberg) and continued until the start of the nationwide CF NBS programme in 2016. However, it should be mentioned that preliminary IRT/PAP trials had already been carried out in the CF NBS centre Dresden since 2005. The IRT/PAP protocol there was performed as originally described by Sarles et al. [8,14], but, as in the Netherlands, the ELISA MucoPAP kit (Dynabio, Marseille, France) was used for PAP quantification, and the photometric detection was replaced by fluorometric measurements [14]. Every year, 18,000 newborns are examined in Dresden and 110,000 in Heidelberg. In Heidelberg, however, less than half of the hospitals that send Guthrie cards to the NBS centre participated in the CF NBS pilot study. The IRT/PAP strategy in Heidelberg has been modified by applying a floating cut-off for IRT using the 99.0th percentile, which is often used in other CF NBS protocols. For PAP, the Heidelberg protocol relied only on one PAP cut-off using the lower PAP cut-off of the two IRT-dependent PAP cut-offs of the original protocol by Sarles et al. [8], which was defined at ≥ 1.67 µg/L (before correction of the dilution factor ≥ 1.0 µg/L) (Figure 1B). In both Dresden and Heidelberg, a safety net strategy was applied from the first year of the study due to ongoing discussions about the possibility of low sensitivity when using PAP. According to this, CF NBS was positive when the IRT ≥ was 99.9 percentile, regardless of the PAP value, which was measured as 2nd tier test. From 2008 until 2016 in Heidelberg, but not in Dresden, a genetic CF NBS protocol searching for the four most common *CFTR* mutations in Germany (IRT/DNA (4)) was run in parallel as a reference.

In 2009, another pilot study was started in the Czech Republic (Prague). In this prospective study 106,522 newborns from Bohemia, the western region of the Czech Republic, were examined to compare the IRT/PAP protocol, as originally published by Sarles et al. [8], with an IRT/DNA/IRT protocol that had been started two years earlier. While for the IRT/PAP protocol the same IRT and PAP cut-offs values were used as originally published, for the IRT/DNA/IRT protocol, the initial IRT was rated positive when the value was ≥ 65 µg/L. The initial DNA test included 32 CFTR mutations, while from July 2010, it contained 50 CFTR mutations, which represented 90.8% and 92.8% of all CFTR mutations of Czech CF patients, respectively. The results of these two protocols were compared and used to simulate an IRT/PAP/DNA(50) protocol, whose performance was then compared to that of the IRT/PAP and IRT/DNA(50)/IRT protocol.

Some of the questions concerning the PAP-based CF NBS protocols could only be answered through cooperation and combinations of study results, as done with those from Heidelberg, Dresden, and Prague. This was the only way to answer questions about the initial IRT cut-off value, the PAP cut-off values, the need for an IRT-dependent safety net, and the performance of a CF NBS strategy using the product of the IRT and PAP values [14,18].

Another PAP-based CF NBS study with 255,000 newborns started in Portugal at the end of 2013 [17]. To the best of our knowledge, this study was the first to test an IRT/PAP/IRT strategy. The cut-off value of the initial IRT was first set at 50 µg/L, but was increased to 65 µg/L after only four months. The second IRT measurement as a third stage strategy was either performed when the initial IRT ≥ was 150 µg/L (SN strategy), the PAP ≥ was 0.5 and the IRT was between 100 and 150 µg/L, or the PAP was ≥ 1.6 µg/L. For PAP analysis, the MucoPAP-F kit (Dynabio, Marseille, France) was used.

## 4. Findings from the Pilot Studies

### 4.1. IRT/PAP Protocols Detect Less Healthy Carriers

The obvious advantage of an IRT/PAP strategy is the complete avoidance of the detection of healthy carriers of *CFTR* mutations by using the pure biochemical parameters IRT and PAP. Interestingly, however, the published results from the Netherlands, Heidelberg (Germany), and the Czech Republic also showed that only 10–20% of newborns who tested positive in IRT/PAP were healthy carriers [11,12,13]. This shows that the heterozygous presence of a *CFTR* mutation alone does not lead to an increased PAP value in the majority of cases, which, in turn, excludes a direct dependence on the presence of certain *CFTR* mutations. This fact may seem unimportant at first glance, but it is of considerable relevance when the decision has to be made in countries with very heterogeneous ethnic populations about whether a genetic or a biochemical CF NBS should be used. While an increased number of *CFTR* mutations in the panel of an IRT/DNA protocol inevitably also increases the number of healthy carriers, a significantly lower detection rate of carriers can be achieved by adding a PAP test prior to the search for *CFTR* mutations. In the pilot study in the Netherlands, the reduction of carriers by the IRT/PAP/DNA(35)/EGA strategy was 88% in comparison to the IRT/DNA (35)/EGA strategy [12].

### 4.2. IRT/PAP Protocols Detect Less CFSPID

The notion that PAP-based CF NBS protocols detect less CFSPID was primarily based on the fact that the first IRT/PAP protocol by Sarles et al., with its two IRT-dependent PAP cut-off levels, was designed in a way that the majority of CFSPID patients are not detected [8]. The reason for using this design was based on the assumption that in the IRT range from 50.0 to 99.9 μg/L, lower PAP values could reflect mild CF phenotypes that are not the goal of CF NBS. As expected, those IRT/PAP protocols showed also in the following pilot studies a significantly lower detection rate of newborns with CFSPID [12,13]. However, so far, there is no evidence that the PAP concentration generally correlates with the severity of CF disease. This fact is also supported by data from the other pilot studies showing higher PAP concentrations in CFSPID or patients with *CFTR* mutations leading to pancreatic sufficiency and low PAP concentrations in some patients with *CFTR* mutations leading to pancreatic insufficiency and a severe CF phenotype (e.g., [18]). When the pilot study on the IRT/PAP strategy was started in Heidelberg in 2008, it was decided that only a single PAP cut-off level of ≥ 1.67 μg/L (before correction of the dilution factor 1.0 µg/L) [11] should be used. Nevertheless, even with this protocol, a significantly lower detection rate for newborns with CFSPID was found. While only 1.6% of the children positively screened by the IRT/PAP protocol with subsequent detection of 2 *CFTR* mutations were newborns with CFSPID, the rate with the IRT/DNA [4] protocol run in parallel was 7.3% [18]. These results indicate that a CF NBS with PAP alone can reduce the detection of CFSPID.

### 4.3. IRT/PAP Protocols May Show Lower Sensitivity than IRT/DNA Protocols

The published pilot studies by Sarles et al. showed that the IRT/PAP strategy had the same—if not better—sensitivity than the IRT/DNA(20/30)/IRT protocol conducted in parallel [8,10]. However, these results could not really be confirmed in any of the other pilot studies (e.g., [12,13,15]). However, it turned out that there may be a variety of reasons for possible reductions of the sensitivity of an IRT/PAP protocol. Several of these drawbacks were addressed in the pilot studies, and it became clear that some of them could be overcome by minor protocol changes. Nevertheless, most of the sensitivity improvements proposed below are at the expense of the PPV, another important quality criterion of CF-NBS protocols.

1. *The use of an IRT-dependent safety net:* When the pilot studies were started in the Germany, the general concern was that the PAP strategy had a worse sensitivity than a well-performing genetic CF NBS. Similar to the IRT/DNA protocols with a restricted mutation panel, an IRT-dependent safety net was added six months after starting the pilot studies. Therefore, CF NBS is considered positive if the initial IRT is above the 99.9th percentile, regardless of the PAP result. When the results of the pilot study conducted in Prague (Czech Republic) were published in 2012, the IRT/PAP strategy showed a very low sensitivity of only 76% [13]. After a re-evaluation for a joint, posthoc analysis of the raw data from Prague, Dresden and Heidelberg, it was found that the sensitivity of the Prague PAP-based CF NBS would have been 89.5% if the colleagues there had used the original IRT/PAP protocol but with the IRT-dependent safety net, as was done in the German centres [18]. Furthermore, a recently published paper on the Dutch CF NBS shows that out of eight CF patients not detected in the IRT/PAP part of the IRT/PAP/DNA(35)/EGA strategy, five would probably have been found if such an IRT-dependent safety net had been used [16].
2. *Renouncing the two IRT-dependent PAP cut-off values:* As mentioned, the reason to use the two IRT-dependent PAP cut-offs was based on the assumption that such a protocol would avoid the detection of CFSPID. In addition, IRT/PAP protocols with two IRT-dependent PAP cut-off values were proposed to detect less healthy newborns as false positives compared to protocols with only one PAP cut-off value. However, the results of the aforementioned joint posthoc analysis of the data from Prague, Dresden, and Heidelberg suggest that IRT/PAP protocols with two IRT-dependent PAP cut-off values may have limited sensitivity compared to those with only one PAP cut-off value. In a joint simulation of raw data from Prague and Heidelberg, it was found that by using two PAP cut-off values, four newborns with two mutations in the *CFTR* gene would have been missed, but would have been detected by the protocol with one PAP cut-off. Only one out of these four newborns carried a *CFTR* mutation with varying clinical consequence and had a normal sweat chloride. The other three newborns were diagnosed with classical CF with pancreatic insufficiency. Two out of these three CF patients suffered from MI and would have been diagnosed clinically. However, the third CF patient would have been missed by all IRT/PAP protocols relying on two IRT-dependent PAP cut-offs [18]. It can be argued whether one has to consider three missed patients with CF or only one, since two out of these three presented with MI.
3. Anyway, the fact that newborns carrying two CF-causing mutations were not detected due to the IRT/PAP protocol with two PAP cut-offs raises the question of whether such a protocol can achieve sufficient sensitivity. It is interesting to note that if the colleagues in Prague had used the same IRT/PAP protocol as that used in Heidelberg, not only with the IRT dependent safety net, but also with only one PAP cut-off value, the sensitivity would have been 94.7%. Also, in a recently published work on the aforementioned Dutch CF NBS program, it was shown that if only one PAP cut-off value had been used, one CF patient out of the eight CF patients not found would still have been detected. With the five CF patients that would have been found by the safety Net, six of the eight CF patients would have been found [16].
4. *Lowering of PAP cut-off values:* Due to the fact that all the pilot studies mentioned above were started with a MucoPAP kit whose PAP cut-off values had not yet been sufficiently evaluated, the most obvious solution for sensitivity problems would have been to simply adjust the PAP cut-off values downwards. Actually, this was also done later by Sarles et al. and reported in a publication in 2014 [10]. However, significantly lowered PAP cut-off values were not only found there, but were seen in recent years also in other PAP-based protocols (e.g., [17]). Yet, it is precisely this approach that significantly increases the number of false-positive newborns detected.
5. *Using both biochemical markers, IRT and PAP, at the same time:* In all current PAP-based CF-NBS protocols, IRT and PAP are used sequentially. However, the simultaneous use of both biomarkers instead of two steps, e.g., by using the product of IRT and PAP, has the potential to make the screening strategy significantly more sensitive than in the IRT/PAP protocols currently in use. Despite the simultaneous use of both parameters, IRT can still be used as a first-tier-parameter that triggers the PAP measurement if it is above a certain cut-off value. Such an approach was demonstrated by the Dresden group in a posthoc analysis using raw data from the pilot studies of the two German CF NBS centres, i.e., Dresden and Heidelberg [14]. The data from Heidelberg showed the highest sensitivity with the IRTxPAP product (98.3%), in contrast to the revised strategy of Sarles et al. published in 2014 (94.9%), and also in contrast to the Heidelberg IRT/PAP-SN protocol (96.6%).
6. *Time-dependent sampling of the dried blood for neonatal screening:* There is unpublished local experience from Australia, still acknowledged by a number of CF NBS specialists, that the use of PAP is not sensitive enough if the dried blood sample for NBS is taken from the infant before the age of 48 h. As a reason for this, it was assumed that the PAP blood levels in infants with cystic fibrosis increase over time. According to our experience, this could be true, but not only in CF infants. In Germany, the collection of the dried blood sample is usually carried out between the 36th and 72nd hour of life, but for special reasons, we sometimes see early or late sampling. If we group all available PAP values of the infants studied in recent years into 12-h intervals, we see a trend of an increase in the 25th, 50th, and 75th percentiles from 24 h to 72 h (personal communication O. Sommerburg). However, when we focused on CF patients not found in our IRT/PAP protocol, we could not confirm that these CF patients were missed because the time of collection of the dry blood sample was before the 48th hour of life. In this regard, after more than 10 years of PAP-based CF NBS, we consider it to be proven that PAP screening with samples collected between 36 and 48 h of life is feasible. Yet, if the majority of infants in a country are screened for NBS before the 36th hour of life, we might imagine that PAP blood levels might still be too low. In this case, we would recommend a comprehensive pilot study to test the feasibility of a PAP-based CF NBS also under these conditions.

### 4.4. Pure Biochemical IRT/PAP Protocols Show a Relatively Low Positive Predictive Value

The reason why no current PAP-based CF-NBS screening program uses a purely biochemical IRT/PAP strategy has to do with the associated low PPV. In various publications from the pilot studies mentioned above, the PPV was stated to be 7.8–15.3% [12,13,14,15]. Furthermore, it is remarkable that almost all of the aforementioned interventions to improve the sensitivity of the PAP step in an IRT/PAP strategy lead to a further reduction of the PPV. However, it should be noted that the disadvantage of a higher false positive rate is compensated for by the expected higher sensitivity. Of note, also a DNA-based protocol, especially with a limited *CFTR* mutation panel, does not guarantee that the required PPV of 30% is reached, as seen with the IRT/DNA protocol run in parallel in the CF-NBS centre Heidelberg (15.3%) and in the French study published 2014 (27.1%) [10,15] (Table 1). However, the combination of a PAP-based two-tier protocol with a third step test such as a search for *CFTR* mutations or a second IRT will maintain the higher sensitivity but eliminate the disadvantage of the lower PPV. This is the reason why all CF NBS protocols currently in use are based on PAP three- or even four-tier strategies. In DNA-based CF-NBS strategies today, extended gene analysis is often used as the 3rd step after the 2nd step was performed with a limited CFTR mutation panel. This strategy also improves both the sensitivity and the PPV of the protocol. However, it does detect significantly more newborns with CFSPID, which is not really desirable. In this respect, a well-performing IRT/PAP/DNA protocol would be superior to a genetic protocol, as described above.

### 4.5. Current PAP-Based CF Screening Protocols in Use

Today, PAP-based CF NS protocols may achieve sufficient performance. One strength of a PAP-based CF NBS is the possibility to use it in multiethnic populations where an appropriate genetic screening is either not possible or is too cost-intensive. Table 1 gives an overview of the performance of PAP-based protocols compared to selected purely biochemical IRT/IRT- or genetic CF NBS protocols. There are currently five European countries where a CF NBS strategy based on PAP is used either in a national or regional setting.

**Figure 2:**
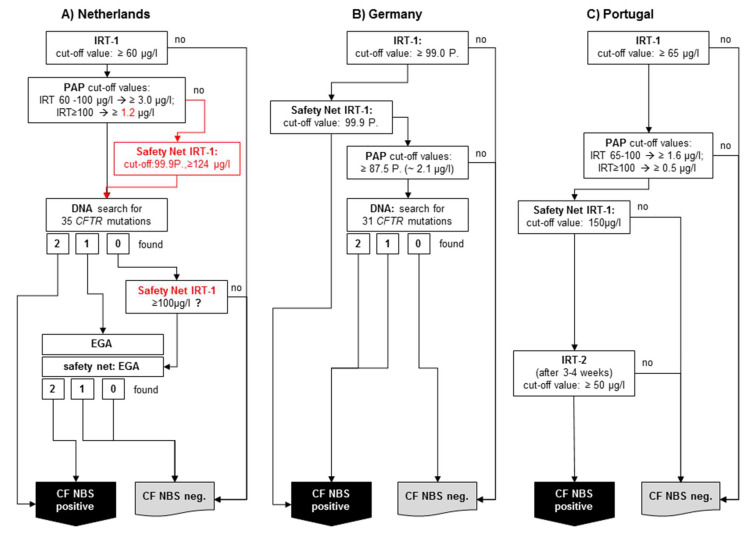
Simplified schemes of three selected PAP-based CF NBS protocols currently used: (**A**) The Netherlands: IRT/PAP/DNA(35)/EGA protocol including last modifications from 2016, (**B**) Germany: IRT/PAP-SN/DNA(31) protocol, (**C**) Portugal: IRT/PAP-SN/IRT protocol.

*The Netherlands:* The first country to use PAP at nationwide level after a pilot study [12] was the Netherlands, which started its national screening program with an IRT/PAP/DNA(35)/EGA protocol in 2011 [16] (Figure 2A). The program started using the commercially-available MucoPAP kit (Dynabio, Marseille, France), but, as mentioned above, the photometric measurement was replaced by a flouroimmunoassay during the pilot study. Until 2016, the IRT/PAP part of the protocol was performed as proposed by Sarles et al. [8], except for the increased IRT cut-off values (now 60 µg/L). However, after the last evaluation published in 2019 [16], the IRT/PAP part of the screening protocol was changed in two points. Firstly, the lower of the two PAP cut-off values was reduced, and secondly, a safety net was introduced for the PAP step, which is based on the 99.9th IRT percentile, as in the protocol according to Sommerburg et al. [11,18]. It may be expected that this variant of the CF-NBS protocol will now have a very high sensitivity and a very good PPV. So far, however, there are no newly-published data on this.

To the best of our knowledge, after the MucoPAP-F became commercially available, it was used for this program. However, it should be noted that in the Netherlands, the two IRT-dependent PAP cut-offs as proposed by Sarles (IRT ≥ 100 µg/L: PAP cut-off ≥ 1.6 µg/L and IRT 60–100 µg/L: PAP cut-off ≥ 3.0 µg/L) were maintained, although it has been recognised that the fluorometric read-out of MucoPAP is higher than that with photometric detection. Nevertheless, this is not a disadvantage for the overall performance. In the genetic part of the protocol, an initial screen will be performed with 35 *CFTR* mutations. Following a different procedure in the past, there is, today, a very comprehensive genetic approach (Figure 2A). All samples showing only 1 *CFTR* mutation and those without mutation but with an IRT > 100 (safety net) receive a very high level extensive gene analysis. Nevertheless, the overall sensitivity of the protocol in the evaluated five years is only 90%, which does not meet the criteria of the ECFS standards of care [19]. The reason for this is clearly the IRT/PAP part and not the DNA (35)/EGA part of the protocol. As shown by Dankert-Roelse et al. 2019 [16] (given also in Table 1), seven CF patients were missed by a low IRT and eight by a low PAP. While problems with a low IRT are difficult to circumvent, the majority of CF patients missed by PAP, as described above, might have been found if a protocol like the one according to Sommerburg et al. [11,18] or Weidler et al. [14] had been used.

In Germany, a PAP-based protocol with a DNA analysis as third tier is also used (Figure 2B). The IRT/PAP-SN part follows the recommendations of Sommerburg et al. 2014, and contains a floating IRT cut-off at the 99.0th percentile and only one PAP cut-off value. Originally, the lower PAP cut-off value (1.6 µg/L) according to Sarles et al. 2005 was used; however, the recommendation is now to apply the 87.5th PAP percentile calculated from PAP values of a nonpreselected population of newborns [25].

After the introduction of the new MucoPAP-F-Kit, the PAP cut-off value, e.g., at the CF NBS centre Heidelberg, is 2.1 µg/L.

If a sample is PAP positive, a search for the 31 most common disease-causing *CFTR* mutations detected by the German national register will be done. If one or two CFTR mutations are found, the sample is rated CF NBS positive. Also, the IRT-dependent safety net (IRT ≥ 99.9th percentile) is used. While samples whose IRT is between 99.0 and 99.9th percentile will be tested for PAP and DNA, samples with an IRT ≥ 99.9th percentile will be immediately rated CF NBS positive [25]. As a reason for this decision, the authorities argued that CF patients whose CFTR mutations were not included in the panel should not be discriminated on the basis of their origin. The expected PPV was calculated in a post hoc analysis and was expected to be 20%, which would not meet the European standards of care [19,26]. This kind of IRT-dependent safety net remains questionable also for other reasons. For example, there is currently no modern CF NBS protocol in which a sample is considered positive after an ultra-high IRT alone. Furthermore, it was shown that, as previously expected, only about 25% of CF patients diagnosed with this protocol received a search for *CFTR* mutations during the CF NBS protocol [26]. Based on data from the Heidelberg IRT/PAP+SN pilot study, the sensitivity of the protocol was estimated to be 96% in the post hoc analysis mentioned above [26]. A complete evaluation of the CF NBS protocol used in Germany is now scheduled to be conducted after 3 years of application.

Portugal started in 2016 with an IRT/PAP-SN/IRT protocol which was evaluated before in the aforementioned pilot study (Figure 2C) [17]. To the best of our knowledge, there are currently no changes in the protocol. The IRT cut-off level was set at 65 µg/L. PAP is measured with the Muco PAP F kit. The PAP cut-off values are IRT dependent: If the IRT value is between 65 and 100 µg/L, a PAP cut-off value of ≥ 1.6 µg/L applies, with an IRT value of ≥ 100 µg/L a PAP cut-off value of ≥ 0.5 applies. Furthermore, an IRT SN strategy (≥150 µg/L) also triggers the measurement of a second IRT (50 µg/L). In our opinion, the PAP cut-off values seem rather low considering the fluorimetric readout of the MucoPAP-F kit used. However, this approach may be advantageous for the sensitivity of the protocol with regard to the multiethnic population in Portugal, especially since the second IRT measurement in IRT/PAP positive neonates will achieve a PPV as required by the European standards. In the pilot study the sensitivity was 94.4% and the PPV 41.03% [17].

In 2017, Austria changed from an IRT/IRT to an IRT/PAP-SN/IRT protocol. PAP measurement is done with the MucoPAP II kit. For the initial IRT, a cut-off value of 65 ng/L was set. The PAP measurement is based on Sarles et al. with two IRT-dependent PAP cut-off values [8,10] that were adapted to the conditions of MucoPAP II: If IRT is between 65 and 100 µg/L, a PAP cut-off value of ≥ 2.5 µg/L applies, if IRT is ≥ 100 µg/L, a PAP cut-off value of ≥ 1.33 µg/L is valid. In addition an IRT-dependent SN (IRT ≥ 130 µg/L) is used. Both an increased PAP and an ultra-high IRT (SN) trigger the second IRT (sampled after 3–4 weeks of age, cut-off value 50 µg/L) [27].

In 2018, Catalonia started using an IRT/PAP-SN/IRT/DNA strategy. PAP measurement is done with the MucoPAP-F kit. The initial IRT cut-off value was set at 50 ng/L. For the second tier, two IRT-dependent PAP cut-off values [8,10] are used, but with other cut-off values, as published elsewhere: If IRT is between 50 and 80 µg/L, a PAP cut-off value of ≥ 1.95 µg/L is used, if IRT is ≥ 80 µg/L, a PAP cut-off value of ≥ 1.0 µg/L applies. An IRT dependent SN with an IRT cut-off value of ≥ 130 µg/L was also implemented in Catalonia. Both an increased PAP and an ultra-high IRT (SN) trigger the second IRT (sampled after 21–30 days of life, IRT cut-off value 35 µg/L). If the second IRT is positive, a comprehensive genetic analysis is performed [28].

Of the PAP-based CF NBS protocols currently used in a national or regional screening programme, only the Netherlands has so far provided performance data of sufficient quality [16]. It is obvious that the data from the other programmes must also be evaluated without delay and the results published. PAP-based protocols definitely have advantages in multiethnic populations, and help to detect less carriers and CFSPID. While the problem of a too low PPV caused by purely biochemical IRT/PAP protocols is probably no longer relevant, as currently, only protocols with at least three tiers are in use, the problem of sufficient sensitivity remains of high relevance.

## Figures and Tables

**Table 1:** Performance indicators sensitivity (%), positive predictive value (PPV, (%)) and CF/CFSPID ratio of a number of representative genetic and PAP-based CF-NBS protocols of different countries and regions compared to the ECFS standard. The numbers in parentheses within the protocol name reflect the CFTR mutations in the panel used.

2nd Tier Test	Reference	Protocol	Region/Country	*n* Screened	Prevalence of CF	Sensitivity (%) w/o MI	PPV (%)
	ECFS standard [19]					≥95	≥30
**IRT**	Calvin et al. 2012 [20]	IRT/IRT	East Anglia (UK)	582,966	1:2286	93.8	67.3
**DNA**	Calvin et al. 2012 [20]	IRT/DNA(29)/IRT	East Anglia (UK)	147,764	1:2111	90.2	85.9
	Sommerburg et al. 2015 [15]	IRT/DNA(4)+SN	Southwest Germany	252,020	1:4582	95.1	15.3
	Kharrazi et al. 2015 [21]	IRT/DNA(28–40)/EGA	California	2,573,293	1:6899	92	34
	Sontag et al. 2016 [22]	IRT/IRT/DNA(41–48)	Colorado, Wyoming, Texas	1,520,079	1:5548	96.2	19.7
	Lundman et al. 2016 [23]	IRT/DNA/EGA	Norway	181,859	1:8660	95	43
	Skov et al. [24]	IRT/DNA(1)/EGA	Denmark	126,338	1:4866	91.7	84.6
**PAP**	Sommerburg et al. 2015 [15]	IRT/PAP+SN	Southwest Germany and East-Saxony (Germany)	328,176	1:4860	96.0	8.8
	Weidler et al. [14]	IRTxPAP	Southwest Germany and East-Saxony (Germany)	410,111	1:5258	97.4	8.2
	Marcao et al. 2018 [17]	IRT/PAP/IRT	Portugal	255,000	1:7500	94.4	41.3
	Dankert-Roelse et al. 2019 [16]	IRT/PAP/DNA(35)/EGA	The Netherlands	819,879	1:6029	90	63
